# Development and Characterization of a Humanized Anti-HER2 Antibody HuA21 with Potent Anti-Tumor Properties in Breast Cancer Cells

**DOI:** 10.3390/ijms17040563

**Published:** 2016-04-15

**Authors:** Ruilin Li, Siyi Hu, Yan Chang, Zhihui Zhang, Zhao Zha, Hui Huang, Guodong Shen, Jing Liu, Lihua Song, Wei Wei

**Affiliations:** 1 Institute of Clinical Pharmacology, Anhui Medical University, Hefei 230032, China; liruilin0986@hotmail.com (R.L.); yychang@ahmu.edu.cn (Y.C.); 2 Key Laboratory of Anti-inflammatory and Immune Medicine, Ministry of Education, Anhui Collaborative Innovation Center of Anti-inflammatory and Immune Medicine, Hefei 230032, China; 3 Department of Pharmacy, the Third Affiliated Hospital of Anhui Medical University, Hefei 230032, China; 4 School of Life Science, University of Science and Technology of China, Hefei 230026, China; husiyi001@163.com (S.H.); zhazhaohanke@hotmail.com (Z.Z.); gdshen@ustc.edu.cn (G.S.); jliu@ustc.edu.cn (J.L.); 5 Anke Biotechnology Co., Ltd., Hefei 230088, China; huanghui0917@gmail.com; 6 Hefei Hanke Mab Biotechnology Co., Ltd., Hefei 230088, China; zhangzhihuidev@126.com

**Keywords:** HuA21, therapeutic antibody, HER2, breast cancer

## Abstract

Human epidermal growth factor receptor 2 (HER2) is one of the most studied tumor-associated antigens for cancer immunotherapy. An engineered anti-HER-2 chimeric A21 antibody (chA21) is a chimeric antibody targeted to subdomain I of the HER2 extracellular domain. Here, we report the anti-tumor activity of the novel engineered monoclonal antibody humanized chA21 (HuA21) that targets HER2 on the basis of chA21, and we describe the underlying mechanisms. Our results reveal that HuA21 markedly inhibits the proliferation and migration of HER2-overexpressing breast cancer cells and causes enhanced antibody-dependent cell-mediated cytotoxicity potency against HER2-overexpressing tumor cells. In particular, HuA21, but not trastuzumab (Tra), markedly suppresses growth and enhances the internalization of the antibody in Tra-resistant BT-474 breast cancer cells. These characteristics are highly associated with the intrinsic ability of HuA21 to down-regulate HER2 activation and inhibit the extracellular signal-regulated kinase 1/2 (ERK1/2) and protein kinase B (Akt) signaling pathways. Furthermore, the combination of HuA21 with Tra synergistically enhances the anti-tumor effects *in vitro* and *in vivo* and inhibits HER2 activation and the ERK1/2 and Akt signaling pathways. Altogether, our results suggest that HuA21 may represent a unique anti-HER2 antibody with potential as a therapeutic candidate alone or in combination with other anti-HER2 reagents in cancer therapy.

## 1. Introduction

Human epidermal growth factor receptor 2 (HER2) is a tyrosine kinase receptor that belongs to the transmembrane epidermal growth factor receptor (EGFR) family, which also includes epidermal growth factor receptor (EGFR/HER1), HER3 and HER4 [1]. HER2 is overexpressed in approximately 10%–20% of breast tumors and plays an important role in tumor development and metastasis [2]. In recent years, a number of monoclonal antibodies and engineered antibody fragments (such as scFvs) targeting HER2 have been developed for tumor diagnosis and therapy [3,4].

The humanized monoclonal antibody trastuzumab (Tra), which specifically targets the extracellular domain of HER2, is approved by the Food and Drug Administration for the treatment of HER2-overexpressing breast cancer [5,6]. Tra blocks HER2 dimerization and inhibits HER2 extracellular signal transduction [7]. These mechanisms include antibody-dependent cell-mediated cytotoxicity (ADCC), the inhibition of downstream substrates, the activation of variety of signaling cascades, including the phosphatidylinositol-3 kinase (PI3K)/serine/threonine-specific protein kinase (Akt) pathway, the induction of cell cycle arrest and apoptosis or the inhibition of tumor angiogenesis [8,9]. Despite the fact that Tra provides clinical benefit to patients with HER2 positive breast cancer, certain patients who initially respond acquire resistance within 24 to 36 months, suggesting the need to develop additional novel therapies targeting HER2 [10]. Furthermore, the most common toxicity of Tra is cardiac toxicity during the course of the treatment [11,12,13]. Another therapeutic antibody pertuzumab, which binds to epitopes within subdomain II of the HER2 extracellular domain showed only partial efficiency in some patients [14]. Several studies show that Tra, in combination with pertuzumab, synergistically increases therapy response rates and improves the survival of patients in HER2-positive early-stage disease patients [15,16].

In recent years, the novel engineered monoclonal antibody anti-HER-2 chimeric antibody 21 (chA21), which is targeted against HER2, was prepared by the surface epitope masking (SEM) method and recognizes a conformational epitope located in subdomain I of the HER2 extracellular domain [12], whereas Tra binds to the juxta-membrane region in subdomain IV [17,18]. Our previous work also established that chA21 inhibits cell proliferation and induces apoptosis in human breast cancer SKBR3 andBT-474 cells *in vitro* and *in vivo* [19,20]. However, the humanized level is approximately 70% because it is a human mouse chimeric antibody. On the other hand, the antigen affinity was only approximately 10 nM, which may affect the clinical cancer treatment effects of the chA21 antibody. Therefore, it is necessary to develop a humanized chA21 antibody that possesses a higher affinity for the antigen of HER2 and a stronger anti-tumor activity. HuA21 was developed by phage display and antibody affinity maturation technologies on the basis of chA21 with a higher affinity, and the degree of humanization was more than 95% [21]. In the present study, we demonstrated that HuA21 treatment induced HER2 and Akt degradation with consequent cell growth inhibition in several models of Tra-resistant cells both *in vitro* and *in vivo*. Meanwhile, the addition of HuA21 also seems to provide significant benefits to the anti-tumor activity of Tra. These data suggest that treatment with HuA21 may represent an effective therapeutic strategy in Tra resistant breast cancer via improving the internalization of antibody and ADCC.

## 2. Results and Discussion

### 2.1. Humanized chA21 (HuA21) Inhibits the Proliferation of Breast Cancer Cells

**Figure 1:**
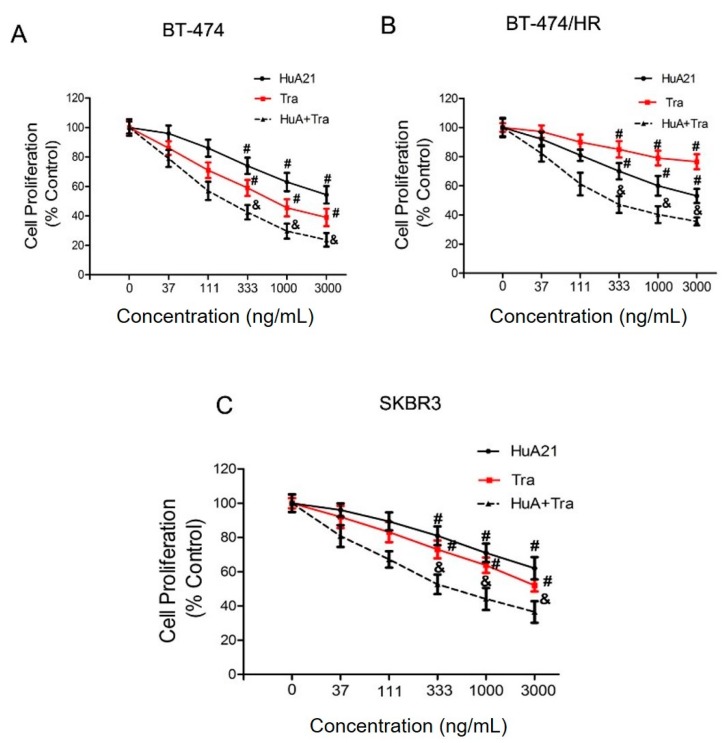
HuA21 suppressed the proliferation of breast cancer cells. The inhibitory effects of HuA21, trastuzumab (Tra), or the combination on the growth of the: BT-474 (**A**); BT-474/HR (**B**); and SKBR3 (**C**) cells. The cells were incubated with HuA21 or Tra at the indicated concentrations for 72 h. Cell viability was measured by the the cell counting kit-8 (CCK-8) assay. The curve charts represent the cell viability of the: BT-474 (**A**); BT-474/HR (**B**); and SKBR3 cells (**C**). The experiments are performed in triplicate, and the data are representative of three separate experiments. # *p* < 0.05 *vs.* control; & *p* < 0.05 *vs.* HuA21-treated cells.

The anti-proliferative effects of HuA21 were analyzed in the BT-474, BT-474/HR, and SKBR3 breast cancer lines. As expected, HuA21 inhibited cell proliferation in the above cell lines in a concentration-dependent manner (Figure 1). The inhibitory ratio of HuA21, Tra, or the combination on the BT-474 cells was 47.22%, 60.89%, and 76.2%, respectively (Figure 1A). HuA21 and the combination, but not Tra, inhibited the proliferation of the BT-474/HR cells as shown in Figure 1B, the inhibitory ratio of HuA21, Tra, or the combination on the BT-474/HR cells was 47.00%, 23.50%, and 64.50%, respectively. In addition, similar to the BT-474 and BT-474/HR cells, HuA21, Tra, or the combination also obviously repressed proliferation of the SKBR3 cells; the inhibitory ratio of HuA21, Tra, or the combination on the SKBR3 cells was 45.50%, 55.20%, and 66.70%, respectively (Figure 1C). These results demonstrate that HuA21 inhibits cell proliferation in breast cancer cell lines.

### 2.2. HuA21 Mediates Antibody-Dependent Cell-Mediated Cytotoxicity (ADCC) against Breast Cancer Cells

**Figure 2:**
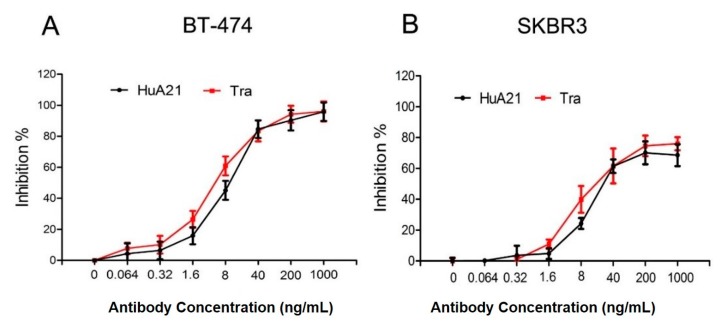
HuA21-mediated ADCC against HER2-positive breast cancer cells SKBR3 (**A**) and BT-474 (**B**) cells were pre-incubated with the indicated concentrations of HuA21 or trastuzumab (Tra). The ADCC experiments were performed in triplicate, and the data are representative of three separate experiments.

ADCC, as opposed to direct HER2 signaling inhibition, is proposed as the major anti-tumor mechanism for Tra [22]. We then tested whether HuA21 mediates ADCC against HER2-positive breast cancer cells. The results showed that HuA21 exerted various degrees of killing against HER2-positive BT-474 and SKBR3 cells. Notably, the HuA21- and Tra-evoked ADCC was similar in the BT-474 and SKBR3 cells, reaching approximately 70% to 96% killing by using an effector/target ratio of 80:1. These results suggested that, like Tra, HuA21 mediates ADCC (Figure 2).

### 2.3. HuA21 Suppressed the Migration of Breast Cancer Cells

**Figure 3:**
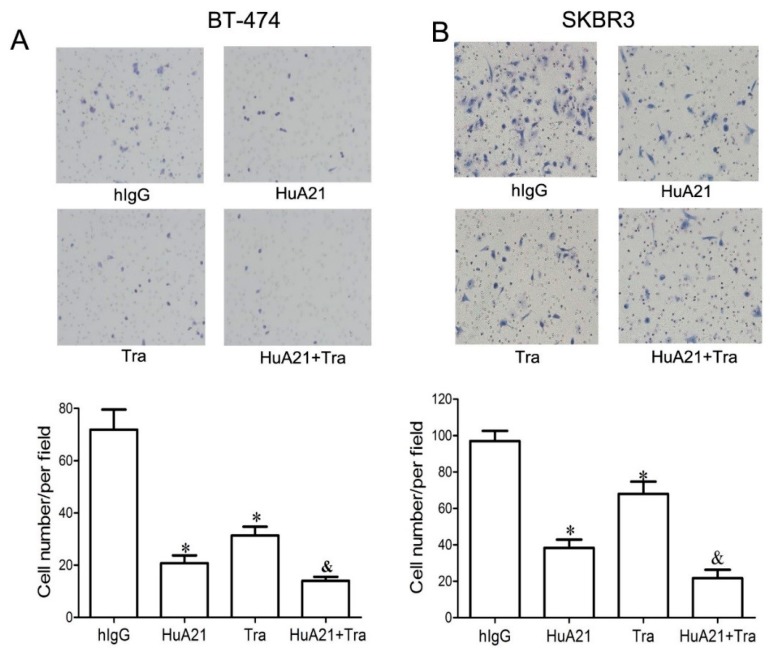
HuA21 suppressed the migration of breast cancer cells. The representative images and bar graph indicate the inhibitory effects of HuA21 (10 µg/mL), trastuzumab (Tra, 10 µg/mL), or HuA21 (10 µg/mL) + Tra (10 µg/mL) on the migration of BT-474 (**A**) and SKBR3 (**B**) breast cancer cells. The cells were treated with HuA21, Tra, or HuA21 + Tra for 24 h. Magnification: 100×. * *p* < 0.05 *vs.* control; & *p* < 0.05 *vs.* HuA21-treated cells. The experiments were performed in triplicate, and the data are representative of three separate experiments.

The effect of HuA21 (10 µg/mL) on the migration of the BT-474 and SKBR3 breast cancer cells was analyzed by a Transwell migration assay. As shown in Figure 3A,B, HuA21 greatly inhibited the migration of the BT-474 and SKBR cells, while less migration inhibition was observed in the Tra-treated BT-474 and SKBR3 breast cancer cells. Meanwhile, in both cell lines, treatment with the combination of HuA21 and Tra resulted in a significantly enhanced inhibitory effect compared with the single agents (Figure 3).

### 2.4. HuA21 Inhibited Human Epidermal Growth Factor Receptor 2 (HER2) Signaling Pathways

**Figure 4:**
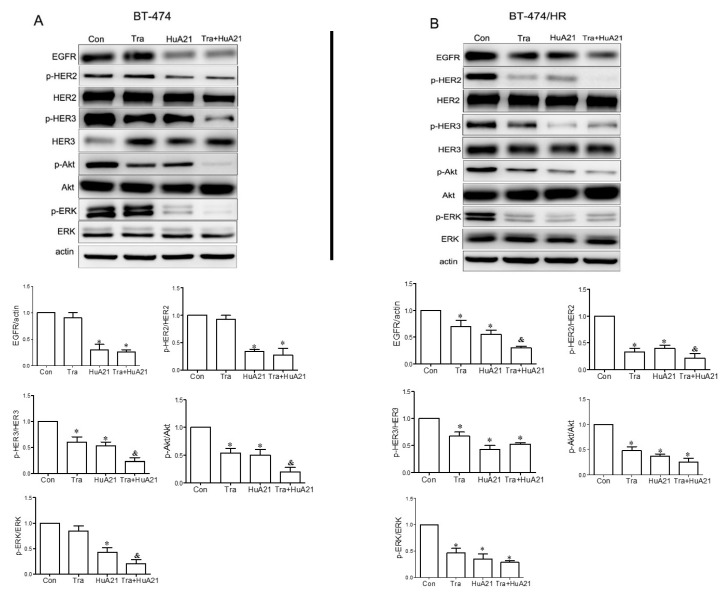
HuA21 inhibited HER2 receptor-related signaling pathways. A Western blot and the representative picture showing the bands of EGFR, p-HER2, total HER2, total HER3, p-Akt, total Akt, p-Erk1/2, and total Erk1/2 expressed in the BT-474 (**A**) and BT-474/HR cells (**B**) treated with HuA21, trastuzumab (Tra), or HuA21 + Tra. β-Actin was used as the loading control. * *p* < 0.05 *vs.* control; & *p* < 0.05 *vs.* HuA21-treated cells. The experiments were performed in triplicate, and the data are representative of three separate experiments.

The MAPK and PI3K-Akt pathways are key signaling pathways of HER2 receptor family-mediated tumor cell proliferation and survival. As shown in Figure 4A,B, in the BT-474 andBT-474/HR cells, both Tra (10 µg/mL) and HuA21 (10 µg/mL) treatment decreased the levels of EGFR, pHER2, and pHER3, and the most obvious decrease in the levels of EGFR, pHER2, and pHER3 was found when the cell were treated with a combination of HuA21 and Tra. Tra, HuA21, or the combination (10 + 10 µg/mL) had little effect on the levels of total HER2 and HER3. An analysis of the downstream signaling pathways revealed that HuA21 and Tra both inhibited receptor downstream signaling, as indicated by the effects on the levels of p-Akt and p-ERK1/2, but the combination of HuA21 and Tra resulted in an enhanced inhibition of signaling as evidenced by the decreased levels of p-Akt and p-ERK1/2. Consistent with the above results, HuA21, but not Tra, suppressed EGFR expression and the phosphorylation of HER2 and HER3 and inhibited p-ERK1/2 and p-Akt expression in the Tra-resistant BT-474/HR cells. Interestingly, the combination of HuA21 and Tra almost completely suppressed the phosphorylation of HER2 and HER3 and EGFR and almost completely inhibited ERK1/2 and Akt phosphorylation.

### 2.5. Internalization Effect of HuA21 in HER2-Positive Cancer Cells

**Figure 5:**
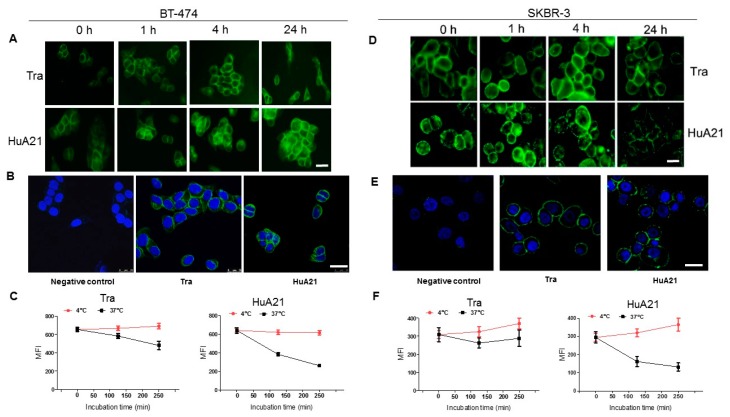
HuA21 enabled internalization in HER2-positive cancer cells. (**A**) The BT-474cells were incubated with FITC-conjugated HuA21 or FITC-conjugated trastuzumab (Tra) for the indicated time periods, and then the presence of HuA21 or Tra was revealed by fluorescence microscope. Representative photomicrographs showing HuA21 or Tra (Green: FITC-conjugated HuA21 or Tra, scale bar = 50 µm); (**B**) The BT-474 cells were incubated with HuA21 or Tra for 4 h, FITC-conjugated secondary antibody was added and the signal was detected by confocal microscopy (Green: FITC-conjugated HuA21 or Tra; nuclei (blue) was stained by 4’,6-diamidino-2-phenylindole (DAPI), scale bar = 50 µm); (**C**) The BT-474cells were incubated with Tra or HuA21 for the indicated periods. HuA21 or Tra on the cellsurface was labeled with a FITC-conjugated secondary antibody and analyzed by flow cytometry; (**D**) The SKBR-3 cells were incubated with FITC-conjugated HuA21 or FITC-conjugated trastuzumab (Tra) for the indicated time periods, and then the presence of HuA21 or Tra was revealed by fluorescence microscope. Representative photomicrographs showing HuA21 or Tra (Green: FITC-conjugated HuA21 or Tra, scale bar = 50 µm); (**E**) The SKBR-3 cells were incubated with HuA21 or Tra for 4 h, FITC-conjugated secondary antibody was added and the signal was detected by confocal microscopy (Green: FITC-conjugated HuA21 or Tra; nuclei (blue) was stained by DAPI, scale bar = 50 µm); (**F**) The SKBR-3 cells were incubated with Tra or HuA21 for the indicated periods. HuA21 or Tra on the cell surface was labeled with a FITC-conjugated secondary antibody and analyzed by flow cytometry. The experiments were performed in triplicate, and the data are representative of three separate experiments.

The effects of HuA21 internalization in HER2-positive breast cancer BT-474 and SKBR3 cells were observed by fluorescence microscopy. Notably, compared with Tra (10 µg/mL), the internalization of HuA21 (10 µg/mL) significantly increased the extended response time in the BT-474 and SKBR-3 cells (Figure 5A,D). HuA21 internalization reached its maximum effect after a 24 h treatment. Using confocal fluorescence microscopy, we further observed the cellular uptake, accumulation and clustering of HuA21 in the cytosolic and perinuclear regions in the two HER2-expressing cell lines (Figure 5B,E). These results showed that HuA21 exhibited a more significant internalization than Tra in the HER2-positive breast cancer cells.

We next measured the residue of HuA21 or Tra on the tumor cell surface by flow cytometry. After treatment for 4 h at 37 °C, the residue of HuA21 or Tra on the BT-474 cell surface was exhibited by 42.2% and 69.5%, respectively (Figure 5C). In addition, in SKBR-3 cells, the residue of HuA21 or Tra on the cell surface was exhibited after incubation for 4 h at 37 °C by 36.1% and 59.4%, respectively (Figure 5F). These results demonstrated indirectly that HuA21 shows a more significant internalization in the two HER2-positive cells.

### 2.6. HuA21 Suppressed Breast Tumor Growth in Vivo

**Figure 6:**
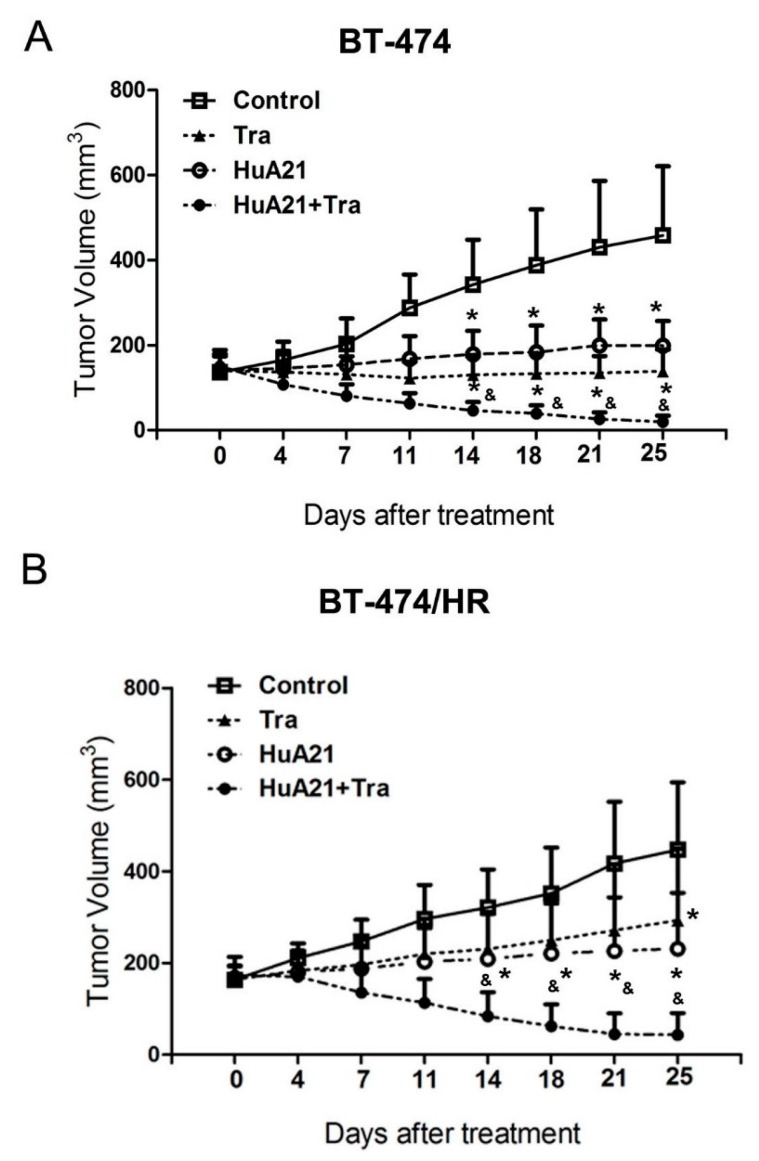
HuA21 suppressed tumor growth *in vivo*. The BT-474 (**A**) or BT-474/HR (**B**) tumor-bearing animals were treated with HuA21, trastuzumab (Tra) or the combination twice per week for three weeks, respectively. The control animal received PBS. The tumor volume of each group was measured at the indicated time periods. * *p* < 0.05 *vs.* the control animals; & *p* < 0.05 *vs.* the HuA21-treated animals, *n* = 8.

To verify the anti-tumor effects of HuA21 *in vivo*, HuA21 was intravenous injected into tumor-bearing mice. The weight of the mice in the treatment groups showed no significant difference (data not shown). The tumor inhibitory rates were 69.8%, 56.6% and 95.8% after Tra, HuA21, and the combination treatment, respectively. All of the treatments significantly repressed tumor growth in the BT474 xenograft tumor model (Figure 6A), and the tumors almost disappeared with the combination treatment. Compared with the BT474 xenograft tumor mice, the administration of Tra in the BT474/HR xenograft tumor mice resulted in a mild inhibition on the tumor proliferation (inhibitory ratio: 34.5%). The inhibitory effect of HuA21 on tumor growth in the BT474/HR xenograft tumor mice was superior to that of Tra (inhibitory ratio: 56.0%). Interestingly, the combination of HuA21 with Tra synergistically enhanced the tumor inhibitory activity (inhibitory ratio: 95.6%) in the BT474/HR xenograft tumor mice (Figure 6B).

### 2.7. Discussion

In a previous study, murine monoclonal antibodies A21 against HER2 were humanized to develop an anti-HER2 chimeric antibody chA21, which inhibits the proliferation and metastasis of HER2 overexpressing human breast cancer cells *in vitro* and *in vivo* [23,24,25,26]. Subsequently, to reduce the potential for generating a human anti-mouse immune response, we developed another novel anti-HER2 engineering-humanized antibody called HuA21 that is based on chA21. In our present study, we explored the anti-tumor activity and the mechanism of the novel anti-HER2 antibody HuA21 *in vivo* and *in vitro*. Our results showed that HuA21 markedly suppressed the proliferation and migration of tumor cells by inhibiting the PI3K/Akt and ERK1/2 signaling pathways *in vitro*. Likewise, our data also showed the therapeutic potential of HuA21 for HER2-overexpressing tumors *in vivo*. The internalization assay showed that a large amount of HuA21 entered into the cells in 1, 4 and 24 h and aggregated to form endosomes. However, Tra was distributed mainly in the cell surface after treatment. The results suggested that HuA21 has a favorable application value in the development of Antibody Drug Conjugates (ADCs).

A significant finding was that the novel anti-HER2 antibody HuA21 not only significantly suppressed the proliferation and migration of the HER2 overexpressing human breast cancer cells but also inhibited cell growth in the Tra-resistant BT-474/HR breast cancer cells. Tra, at a similar concentration, had a comparable anti-proliferative effect on the HER2 overexpressing human breast cancer cells, but no significant inhibitory effect was observed in the HER2/HR cancer cells at our present conditions. Meanwhile, although a similar efficacy of the ADCC of HuA21 and Tra in the breast cancer cells was found, a markedly higher HuA21 internalization in the HER2-positive cancer cells was observed. Tra is believed to exhibit relatively weak effects in inducing HER2 receptor internalization and down-regulation *in vitro* and *in vivo*, which is consist to previous reports by several other studies [27,28]. Simultaneously, ADCC likely leads to an increase in the therapeutic efficacy of HuA21. In addition, HuA21 treatment also showed a stronger inhibitory effect on the migration of HER2-positive breast cancer cells *in vitro*. A combination of HuA21 with Tra further enhanced the inhibition of migration on the HER2-positive breast cancer cells. Furthermore, the *in vivo* assay showed that HuA21 treatment significantly inhibited the tumor growth in both the HER2-overexpressing BT474 xenograft tumor and in the BT474/HR xenograft tumor. The BT474/HR xenograft tumor is resistant to Tra [29]. Tra only suppressed the growth of the BT474 xenograft tumor, but not the BT474/HR xenograft tumor. Intriguingly, in combination with Tra, HuA21 showed a greater benefit for the suppression of tumor growth compared to HuA21 or Tra treatment alone *in vivo*. A possible explanation for this could be the different internalization rates of the antibodies. The degree of internalization of HuA21 was much higher than Tra. Another possible reason could be the different surface epitopes between HuA21 and Tra. HuA21 recognizes subdomain I of the HER2 extracellular domain [12], and Tra binds to the juxta-membrane region in subdomain IV [17,18]. The final possible reason could be the synergistic anti-tumor activities of the combination of HuA21 with Tra, which is consistent with a previous study showing that combinations of anti-HER2 antibodies with different epitopes synergistically down-regulate HER2 and enhance anti-tumor effects [25,30,31]. A more detailed mechanistic explanation of HuA21 should be addressed in future studies.

HER2-mediated activation of the MAPK and PI3-kinase/Akt pathways is related to tumor cell proliferation and metastatic potential [32]. Meanwhile, the sustained activation of MAPK and Akt are believed to be involved in Tra resistant cancer [33,34]. In the present study, our results show that Tra and HuA21 both, not only significantly downregulated HER2 and HER2 phosphorylation but also prevented the phosphorylation of Akt and ERK1/2 in the HER2-overexpressing BT-474 cells. Meanwhile, HuA21, but not Tra, downregulated HER2 and HER2 phosphorylation and inhibited the phosphorylation of Akt and ERK1/2 in the Tra-resistant cancer cells. Similar to the results demonstrating the effects of the combination of HuA21 with Tra on the proliferation and migration of the breast cancer cells and the growth of tumor xenografts, a combination of HuA21 with Tra also enhanced the inhibition the phosphorylation of both Akt and ERK1/2 and reduced HER2 expression and HER2 phosphorylation. Therefore, combining HuA21 with Tra might lead to increased anti-tumor effects.

## 3. Materials and Methods

### 3.1. Materials

Dulbecco’s modified Eagle’s medium (DMEM) medium, Roswell Park Memorial Institute-1640 (RPMI 1640), and fetal bovine serum (FBS) were from GIBCO-BRL (Thermo Fisher Scientific, Waltham, MA, USA). Antibodies against total- and phosphor (p)-Akt (Ser^473^), total- and p-extracellular signal-regulated kinase 1/2 (ERK1/2) (Thr^202^/Tyr^204^), EGFR, HER2 and p-HER3 (Tyr^1289^) were purchased from Cell Signaling Technology (Danvers, MA, USA). Antibodies against β-actin and HER3 were obtained from Santa Cruz Biotechnology (Santa Cruz, CA, USA). The antibody against p-HER2 was from Neomarkers. The horseradish peroxidase (HRP)-conjugated goat anti-mouse and HRP-conjugated goat anti-rabbit antibodies were from Pierce (Pierce, Rockford, IL, USA). The cell counting kit-8 (CCK-8) was purchased from Dojindo (Dojindo, Kumamoto, Japan). Tra was purchased from Roche, and the humanized monoclonal antibody HuA21 was prepared by our research group as described in a previous study [23].

### 3.2. Cells Culture

The human breast cancer cells lines BT-474 and SKBR3 were obtained from the American Type Culture Collection (Rockville, MD, USA). All of the tumor cell lines were cultured in Roswell Park Memorial Institute (RPMI) 1640 containing 10% fetal bovine serum (FBS), 100 U/mL penicillin, 100 µg/mL streptomycin and 2 mM glutamine (all from Thermo Fisher Scientific) in 5% CO_2_ at 37 °C.

### 3.3. Trastuzumab (Tra) Resistant BT-474 Cells (BT-474/HR)

BT-474 resistant breast cancer cells were developed by culturing the cells in the presence of 100 µg/mL Tra for six months. Briefly, BT-474 was initially treated with Tra (10 µg/mL). When the cells reached 80% confluence, the concentration of Tra was changed to 20 µg/mL. Repeating the same procedure mentioned above, the concentration of Tra was changed to 50 and 100 µg/mL in sequence. Finally, the Tra resistant BT-474 cells (BT-474/HR) were obtained. An estrogen pill (0.72 mg/kg) was embedded into nude mice subcutaneously. Three days later, these cells were collected and resuspended at a density of 1.0 × 10^8^/mL and then mixed with Matrigel (BD Biosciences, San Jose, CA, USA, and volume ratio 1:1). A mixture of the cells with Matrigel was injected into the second breast pad of the nude mice. The female nude mice were treated with 10 mg/kg Tra via an intravenous injection once a week for 4 weeks after tumor stabilization. Then, the primary cells were separated and cultured in the presence of 100 µg/mL until the stable resistant cells were generated. The resistance of the cells to Tra was verified by cell viability assays. Compared to the BT-474 cells, the effect of Tra on the inhibition of cell growth of the BT-474/HR cells was markedly reduced (Supplementary Figure S1).

### 3.4. Cell Proliferation Assay

The BT-474, BT474/HR (2 × 10^3^ cells/well), and SKBR3 cells (2 × 10^4^ cells/well) were seeded into 96-well plates and serum-starved for 24 h. Cell proliferation was evaluated using a CCK-8 kit (Dojindo, Japan) according to the manufacturer’s protocol. Briefly, the cells in the 96-well plates were incubated with different concentrations of Tra or HuA21 for 96 h, the CCK-8 solution (10 µL) was added to each well, and the samples were incubated at 37 °C for 1 h before the absorbance was measured at 490 nm. The data are from at least three independent experiments and each experiment was performed in duplicate.

### 3.5. ADCC Assay

ADCC activity was assessed by the method previously described [35]. A standard lactate dehydrogenase (LDH) release assay was used to calculate the BT-474 or SKBR3 cells in the presence of the peripheral blood mononuclear cells (PBMC) (RosetteSep™ Human NK Cell Enrichment Cocktail; Stem Cell Technologies, Vancouver, BC, Canada) isolated from fresh whole blood obtained from a healthy donor at an effector: target ratio of 80:1/well. In brief, aliquots of 0.1 mL of breast cancer target cells (2 × 10^4^ cells) were dispensed into round-bottomed tubes, and 0.1 mL of the effector cells was added to the target cells to give a desired effector-to-target (E:T) cell ratios. The indicated concentrations of Tra and HuA21 were added and were incubated for 5.5 h. The LDH release was evaluated by measuring absorbance at 490 nm on a Multiskan Ascent 96-well plate reader (Thermo Fisher Scientific).

### 3.6. Cell Migration Assays

Cell migration was evaluated in a modified Boyden chamber of 24-wells plates, containing transwell inserts (polycarbonate membrane insert with 6.5-mm diameter and 8.0 µm pores; Corning, Schiphol, The Netherlands). As a conditional cultivation liquid, 10% FBS was added to the lower chamber of the polymerized Transwell chambers in each group and 100 µL of the cell suspension was added to the upper chamber and was incubated for 24 h, fixed using 4% paraformaldehyde and stained with a 0.25% hematoxylin solution (Tianjin Chemical Reagent Factory, Tianjin, China). The cells in the lower chambers were counted under a light microscope (magnification, 200×). A total of five transmembrane cells in randomly selected visual fields were counted separately on each membrane and the average was calculated. There were three chambers in each group with three duplicates.

### 3.7. Immunoblotting Analysis

The cells in each group were collected. The total protein was extracted and its concentration was measured using a bicinchoninic acid assay (Thermo Fisher Scientific). The total protein (30 µg) was separated by a 10% sodium dodecyl sulfate (SDS) polyacrylamide gel electrophoresis (PAGE; Gibco-BRL, Carlsbad, CA, USA) and was electrotransformed onto a polyvinylidene difluoride membrane. The membranes were blocked with 5% non-fat milk and were then incubated with a primary antibody at 4 °C overnight. An HRP-conjugated secondary antibody and an HRP-conjugated Actin were added to the membrane, which was incubated at 37 °C for 2 h. The immunoreactive proteins were visualized by enhanced chemiluminescence, and the signal intensity was detected and quantified by Image Quant LAS4000mini (GE Health Care, Pittsburgh, PA, USA).

### 3.8. Internalization Assays

The internalization of the antibodies was evaluated by fluorescence microscope, confocal fluorescence microscope (Leica TCS SP8, Mannheim, Germany), and flow cytometry (Becton Dickinson, NJ, USA), respectively. For immunofluorescence, the SKBR3 or BT-474 cells were seeded on coverslips. After 24 h, the cells were incubated with FITC-conjugated HuA21 for 1, 4 and 24 h. The cells were placed in formalin overnight, and after washing with ice-cold PBS, confocal images were taken. The cells were fixed in 4% paraformaldehyde for 30 min at room temperature. Immunostaining was performed using a HuA21 antibody (dilution in 1:100) and a FITC-conjugated second antibody (1:1000; Thermo Fisher Scientific), and the cells were counterstained for nuclei with DAPI.

For the flow cytometry analysis of endocytosis, the plated cells were incubated at 37 °C in complete culture medium. After washing in PBS, the SKBR3 or BT-474 cells were recovered by a trypsin- ethylene diamine tetraacetic acid treatment and were incubated for another 30 min at 4 °C in the presence or in the absence of the indicated antibodies (1–2 µg/mL), and the cell-associated fluorescence was determined by flow cytometry. To look for the specific uptake, the cells were incubated for 2 h at 37 °C in the presence of a fluorescein-labeled conjugate. Cell-associated fluorescence was assessed using a BD-LSR flow cytometer (BD Biosciences), and the data were analyzed with Cell Quest software (BD Biosciences). The gates used for the flow cytometric analysis were shown in Supplementary Figure S2.

### 3.9. Tumor Xenografts

The animal experiment was ethically approved by the Laboratory Animal Ethics Committee in the Institute of AnHui Medical College. All of the experimental procedures were performed in conformity with the institutional guidelines and protocols for the care and use of laboratory animals. Briefly, an estrogen pill was embedded into the nude mice subcutaneously. Three days later, the BT474 and BT474/HR cells were collected and resuspended at a density of 1.0 × 10^8^/mL and then mixed with Matrigel (BD Biosciences, volume ratio 1:1). A mixture of the cells with Matrigel was injected into the second breast pad of the nude mice. The BT-474 and BT-474/HR tumor-bearing animals were randomized into four groups: control; Tra (10 mg/kg); HuA21 (10 mg/kg); and the combination of HuA21 (5 mg/kg) with Tra (5 mg/kg). All of the treatments were given via an intravenous injection twice weekly for 3 weeks. The control animal was received PBS. The mice were sacrificed to measure the tumor weight after the experimental time periods. The tumor inhibitory rate = (Volume of the control group − Volume of the treated group)/Volume of the control group × 100%.

### 3.10. Statistical Analysis

The results are expressed as the mean ± standard error of the mean. All of the values for each treatment group were normalized to the un-stimulated cells. All of the data analysis was performed with the use of GraphPad Prism 5 software (La Jolla, CA, USA). Differences between the mean values of multiple groups were analysed by a one-way analysis of variance with Tukey’s test for *post hoc* comparisons. Statistical significance was considered at *p* < 0.05.

## 4. Conclusions

In conclusion, our study demonstrates that the novel anti-HER2 humanized antibody HuA21 exerts anti-tumor activity *in vitro* and *in vivo* via enhanced antibody internalization and the inhibition of the HER-related ERK1/2 and Akt pathways. Meanwhile, a combination of HuA21 with Tra synergistically enhances the anti-tumor effects of Tra, which may be mediated by the HuA21 down-regulation of HER2 expression as well as an interruption of downstream signals. Therefore, HuA21 might represent an anti-HER2 antibody with superior potential for future anti-tumor therapy.

## Supplementary Materials

Supplementary materials can be found at http://www.mdpi.com/1422-0067/17/4/563/s1.

## Abbreviations

ADCC	antibody-dependent cell-mediated cytotoxicity
ADCs	Antibody Drug Conjugates
DMEM	Dulbecco’s modified Eagle’s medium
EGFR	epidermal growth factor receptor
ERK1/2	extracellular signal-regulated kinase 1/2
HER2	human epidermal growth factor receptor 2
LDH	lactate dehydrogenase
PI3K	phosphatidylinositol-3 kinase
