# Supplementary material for: Development and Characterization of a Humanized Anti-HER2 Antibody HuA21 with Potent Anti-Tumor Properties in Breast Cancer Cells
Source: Int J Mol Sci. 2016 Apr 15;17(4):563. doi: 10.3390/ijms17040563 (PMC4849019; doi:10.3390/ijms17040563)
Supplement: Supplementary file 1 [file ijms-17-00563-s001.pdf]

Supplementary Materials: Development and Characterization of a Humanized Anti-HER2 Antibody HuA21 with Potent Anti-Tumour Properties in Breast Cancer Cells

Ruilin Li, Siyi Hu, Yan Chang, Zhihui Zhang, Zhao Zha, Hui Huang, Guodong Shen, Jing Liu, Lihua Song and Wei Wei

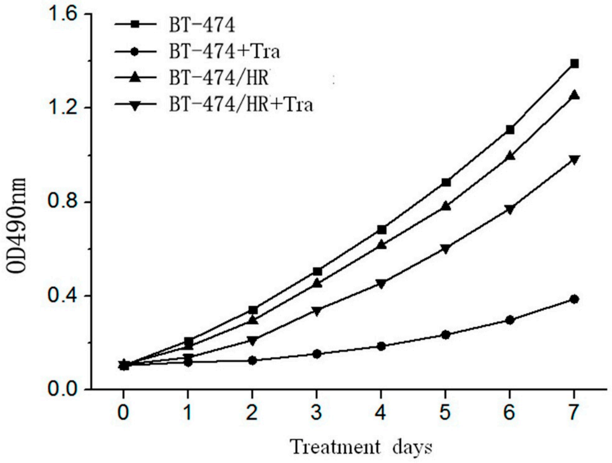

Figure S1. The effects of trastuzumab (Tra) on proliferation of BT-474 and BT-474/HR cells *in vitro*. The BT-474 and BT-474/HR cells was incubated with or without trastuzumab (10 μg/mL) for indicated periods, the proliferation was measured by a CCK-8 proliferation kit.

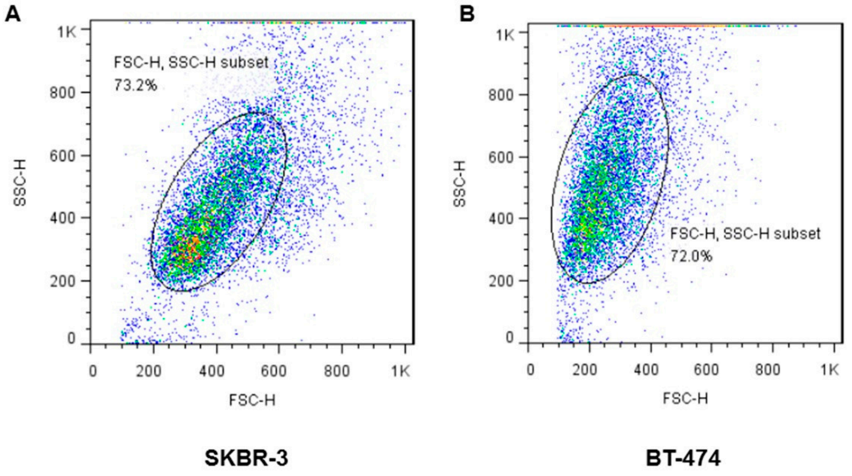

Figure S2. The gates were used for the flow cytometric analysis for the two tumour cells. (**A**) The gate was used for the flow cytometric analysisfortheSKBR3 cells; (**B**) The gate was used for the flow cytometric analysisfortheBT-474 cells. The blue marked cells mean uninternalized antibody cells, while the other colored cells represent FITC-conjugated HuA21.
